# Global, Regional, and National Burden of Autism Spectrum Disorder: Trends and Decomposition Analysis From 1990 to 2021, and Projections for 2045

**DOI:** 10.62641/aep.v53i6.2029

**Published:** 2025-12-17

**Authors:** Huihui Zhu, Wenjing Ye, Fan Zhou, Linwei Shi, Yonghai Zhou, Peining Liu

**Affiliations:** ^1^Department of Child Health Care, The Second Affiliated Hospital and Yuying Children’s Hospital of Wenzhou Medical University, 325000 Wenzhou, Zhejiang, China

**Keywords:** global burden of disease, Socio-demographic Index, autism spectrum disorder, disability-adjusted life years, estimated annual percentage change

## Abstract

**Background:**

Autism Spectrum Disorder (ASD) has rising global prevalence and lifelong impacts. We quantified its 1990–2021 burden using Estimated Annual Percent Change (EAPC) trends, decomposition analysis, and the Nordpred model to project burdens to 2045.

**Methods:**

This study analyzed the global, regional, and national ASD burden from 1990 to 2021 using the Global Burden of Disease (GBD) 2021 database, assessing prevalent cases, Disability-Adjusted Life Years (DALYs), Age-Standardized Prevalence Rate (ASPR), and Age-Standardized Disability-Adjusted Life Years Rate (ASDR). It used EAPC to analyze trends, decomposition analysis to examine contributors, and the Nordpred model for predictions to 2045.

**Results:**

From 1990 to 2021, prevalent cases rose from 41,929,995.80 to 61,823,539.64, males more affected. ASPR increased from 773.25 to 788.34/100,000, ASDR from 144.51 to 147.56/100,000. The number of DALYs increased by 3.68 million (95% Uncertainty Interval 3.20–4.10 million) from 1990 to 2021. Middle Socio-demographic Index (SDI) regions saw the largest increases, High SDI regions minimal growth. Among 21 GBD regions, high-income Asia Pacific grew fastest, Oceania declined. Nationally, Japan, South Korea, and Singapore had the highest 2021 ASPR/ASDR, Bangladesh, Brazil, and Nepal the lowest. Decomposition analysis showed population growth drove prevalent cases and DALYs increases, aging caused declines. Predictive models estimate 71,782,946 cases by 2045, DALYs peaking at 13,365,467 years. ASPR and ASDR expected to peak in 2029 (792.16/100,000) and 2034 (148.55/100,000), then decline.

**Conclusion:**

The rising ASD burden requires immediate action, particularly in middle SDI regions and high-income Asia Pacific, where growth is speeding up. Early intervention and equitable resource distribution for high-risk groups like males and fast-growing populations are essential to cut projected case and DALY increases by 2045.

## Introduction

Autism spectrum disorder (ASD) is a group of neurodevelopmental disorders characterized primarily by social communication impairments, repetitive stereotyped behaviors, and restricted interests [1]. Since Leo Kanner first described autism in 1943 [2], research in this field has advanced rapidly. The prevalence of ASD has been increasing annually, making it a globally recognized public health issue. Since the earliest epidemiological surveys in the 1960s, a wealth of data has become available, indicating that the prevalence of the disorder is much higher than previously expected [3]. Early studies from the 1960s to 1980s reported a conservative estimate of 1 to 5 cases per 10,000 individuals [4, 5, 6]. This figure rose to a median prevalence of 62 per 10,000 (0.62%) for the ASD according to systematic reviews in 2012, equating to approximately 1 in 160 children worldwide [7]. This trend has continued its upward trajectory. Data from the U.S. Autism and Developmental Disabilities Monitoring (ADDM) Network illustrate this increase, with prevalence among 8-year-old children rising from 1 in 110 in 2006 to 1 in 44 by 2022 [8]. Recent national data from the United States reports an overall ASD prevalence of 3.19% among school-aged children. Among these cases, an estimated 10.1% are characterized by severe symptoms. This subgroup often presents with co-occurring intellectual disability (a condition that affects 40–60% of the broader ASD population) and requires substantial support [9]. The pathogenesis of ASD remains unclear, and there is currently a lack of effective pharmacological treatments. Management primarily relies on rehabilitation therapies, with pharmacological treatments playing a supplementary role. ASD is a chronic condition with high treatment costs yet limited efficacy [10]. The high costs associated with ASD are largely due to the special education costs during childhood and the costs related to housing, healthcare, and productivity losses in adulthood [11, 12]. Although autism symptoms generally improve with age, social integration remains poor [13].

The progress made in comprehensive treatment programs for very young children, if extended to intervention measures throughout the entire life cycle, could lead to a more positive future for children with autism growing up today, according to Piven J *et al*. [14]. Therefore, epidemiological surveys are considered a priority, as their importance lies not only in providing objective and reliable estimates of prevalence but also in helping to assess the specific needs and priorities of each region. While previous studies have documented the rising prevalence and burden of ASD, few have projected its long-term trajectory or quantified the contributions of demographic and epidemiological drivers. Moreover, no study to date has predicted the global burden of ASD up to 2045, highlighting a critical gap in the literature.

In this study, we aim to address this gap by comprehensively evaluating the global burden of ASD from 1990 to 2021 and projecting future trends to 2045. We will further quantify the contributions of key demographic factors to the changes in ASD burden. This study aims to provide more precise data support and strategic recommendations for the global epidemiology of autism, contributing to the reduction of disease burden and the maintenance of public health.

## Methods

### Data Source 

The Global Burden of Disease (GBD) 2021 study is an international collaborative project led by the Institute for Health Metrics and Evaluation (IHME). Its primary objective is to systematically quantify and compare the health impacts of diseases, injuries, and risk factors across age, sex, and geographical groups [15]. Since 1990, the study has annually assessed the global burden of disease using a consistent methodology [15]. The Socio-demographic Index (SDI), ranging from 0 to 1, is used to classify regions into five developmental levels: low (<0.46), lower-middle (0.46–0.60), middle (0.61–0.69), upper-middle (0.70–0.81), and high (>0.81) [16]. The scope of GBD 2021 is extensive, covering 204 countries and territories, 21 GBD regions, and incorporating 371 diseases and injuries as well as 88 risk factors. The study provides detailed estimates of incidence, prevalence, mortality, years of life lost (YLL), years lived with disability (YLD), and disability-adjusted life years (DALYs) [15].

Based on the GBD 2021 database, we collected global, regional, and national epidemiological data on ASD across all age groups from 1990 to 2021, including the number of prevalent cases, DALYs, Age-Standardized Prevalence Rate (ASPR), and Age-Standardized Disability-Adjusted Life Years Rate (ASDR). The data were organized into 5-year age groups, providing essential support for a comprehensive analysis and in-depth understanding of the disease burden and temporal trends of ASD. All data used in this study is publicly accessible through the GBD Results Viz Hub (https://vizhub.healthdata.org/gbd-results/).

### Case Definition for ASD

The GBD 2021 study defines ASD using International Classification of Diseases, 10th Revision (ICD-10) codes F84.0–F84.9 [17]. While aligning with modern diagnostic frameworks, prevalence trends are notably influenced by evolving diagnostics and surveillance heterogeneity, particularly in low-to-middle SDI countries lacking gold-standard tools. To address this, the GBD model employs: (1) statistical crosswalking for data harmonization; (2) meta-regression (DisMod-MR) using covariates like SDI to estimate prevalence in data-sparse areas [18]; and (3) quantified uncertainty with wider intervals for less reliable data [19]. This approach separates true epidemiological changes from diagnostic variations, with non-etiological influences captured within our ’epidemiological changes’ component.

### Statistical Analysis

#### Trend Analysis Using EAPC

To analyze the trends in ASPR and ASDR for ASD, we employed the Estimated Annual Percentage Change (EAPC) method. The formula for EAPC is: *EAPC = 100 × [exp(β) – 1]*, where β is the slope obtained from the linear regression model ln (*Age-Standardized Rate (ASR*)) = α + βX + *e*. Here, ln(*ASR*) represents the natural logarithm of ASR, X is the year, α is the intercept, and *e* is the random error. The trend is determined based on the EAPC and its 95% confidence interval (CI): if both the EAPC and the lower limit of the CI are positive, the trend is considered to be increasing; if both the EAPC and the upper limit of the CI are negative, the trend is considered to be decreasing; if neither condition is met, the trend is considered stable [20].

#### Decomposition Analysis

To assess the impact of population aging, population growth, and epidemiological changes on ASD, we applied the decomposition analysis method proposed by Das Gupta [21]. Using this method, we decomposed the changes in the number of ASD cases and DALYs from 1990 to 2021 into three main demographic determinants: population aging, population growth, and epidemiological changes. The “epidemiological changes” component captures variations due to diagnostic criteria updates, improvements in surveillance, and shifts in awareness and reporting practices. This approach quantifies the contribution of each factor to the overall change, helping us better understand how these factors collectively influence the burden of ASD.

#### Prediction Model

To predict trends from 2022 to 2045, we applied the Nordpred Age-Period-Cohort model [22]. The analysis used 20 five-year age groups (<5 to 95-plus). Annual data from 1990–2021 were aggregated into 5-year periods for modeling. Key parameters defining the prediction intervals were specified: the net drift was estimated using a joinpoint model (cuttrend = c(0, 0.25, 0.5, 0.75, 0.75)), applying weights to minimize instability in extreme ages. The model used a power-5 link function (linkfunc = “power5”) and assumed a Poisson distribution (no over-dispersion correction). The predictions are presented as line graphs at 5-year intervals from 2022 to 2045, with 95% prediction intervals to evaluate the uncertainty of the forecasts.

#### Software and Implementation

Statistical analyses were conducted using R (version 4.3.3; R Foundation for Statistical Computing, Vienna, Austria). Data visualization was performed with the ggplot2 package (version 3.5.2; Posit, PBC, Austin, TX, USA). Final figures were assembled and refined using Adobe Illustrator 2024 (version 28.4; Adobe Inc., San Jose, CA, USA).

## Results

### Global Level

**Fig. 1.**
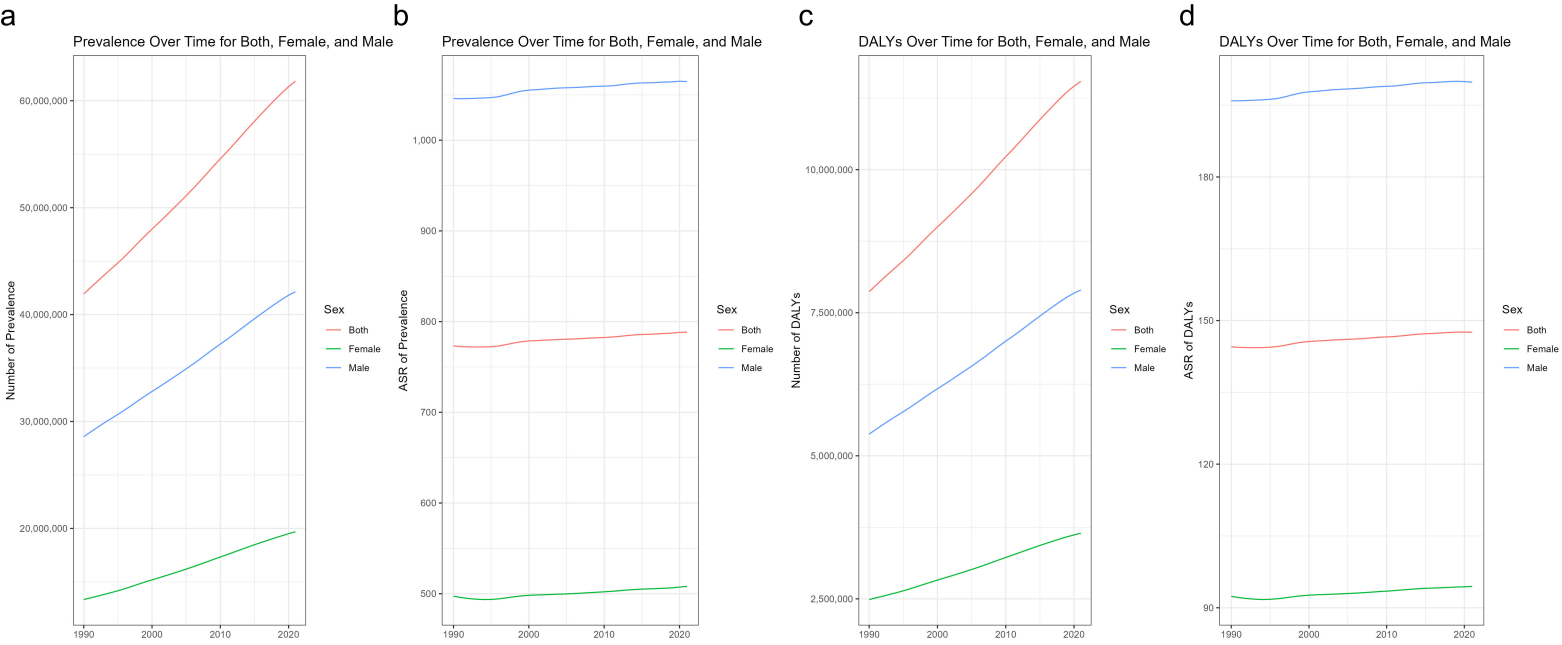
**Global Trends of ASD Prevalence and DALYs from 1990 to 2021**. Number of Prevalence (a), ASR of Prevalence (b), Number of DALYs (c), and ASR of DALYs (d). ASD, autism spectrum disorder; DALYs, Disability-Adjusted Life Years; ASR, Age-Standardized Rate.

**Fig. 2.**
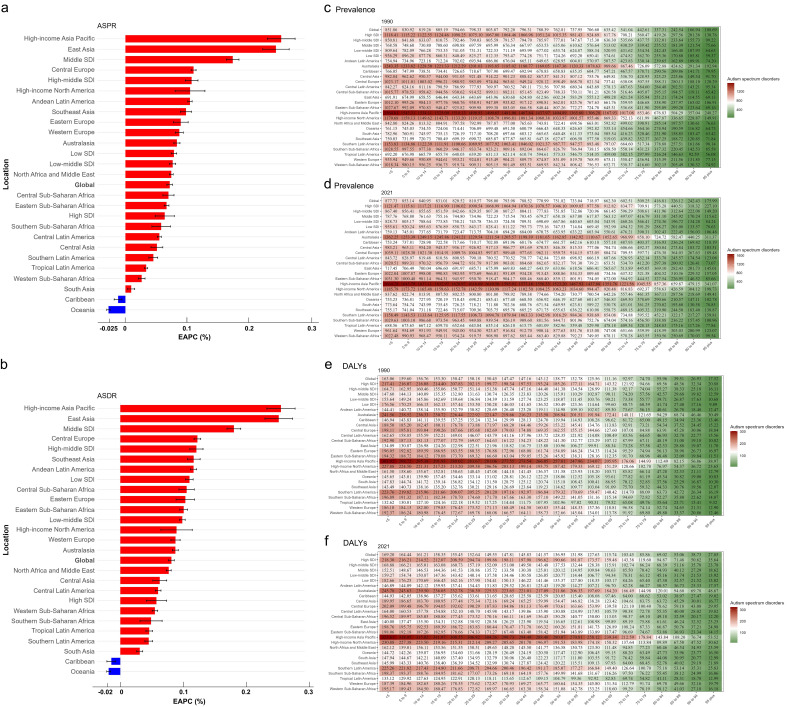
**Global and Regional EAPCs of ASD, and Rates of Prevalence and DALYs**. EAPC for ASD Prevalence (a) and DALYs (b) from 1990 to 2021. ASD prevalence and DALYs rates across age groups for 1990 (c,e) and 2021 (d,f), by global, five SDI, and 21 GBD regions. EAPC, Estimated Annual Percent Change; ASD, Autism Spectrum Disorder; SDI, Socio-demographic Index; GBD, Global Burden of Disease; DALYs, Disability-Adjusted Life Years; ASDR, Age-Standardized Disability-Adjusted Life Years Rate; ASPR, Age-Standardized Prevalence Rate.

From 1990 to 2021, both the number of prevalent cases and the ASPR of ASD increased globally. Throughout this period, males consistently exhibited higher ASPR and greater numbers of prevalent cases compared to females (Fig. 1a,b; Tables 1,2). Similarly, DALYs and the ASDR also rose, with males experiencing a higher burden than females (Fig. 1c,d; **Supplementary Tables 1,2**). The EAPC in ASPR was 0.072 (95% CI: 0.067 to 0.076) globally, with a more pronounced increase among females (EAPC = 0.090) than males (EAPC = 0.064). The ASDR also increased, with an EAPC of 0.081 (95% CI: 0.076 to 0.086), again higher in females (EAPC = 0.098) than males (EAPC = 0.074) (Fig. 2a,b; **Supplementary Fig. 1**; Table 2,** Supplementary Table 2**). In both 1990 and 2021, the highest prevalence and DALY rates were observed in the under-5 age group, while the lowest were in the 95-plus age group (Fig. 2c–f).

**Table 1.** **Number of prevalent cases of autism spectrum disorder (ASD) by location and sex in 1990 and 2021**.

Location	Prevalence			Prevalence		
	1990			2021		
	Both	Male	Female	Both	Male	Female
Global	41,929,995.80	28,580,940.33	13,349,055.47	61,823,539.64	42,133,878.51	19689661.13
High SDI	8,972,557.02	6,213,740.38	2,758,816.64	11,056,985.96	7,733,412.70	3,323,573.25
High-middle SDI	8,301,430.47	5,764,488.19	2,536,942.28	10,045,485.86	7,142,197.26	2,903,288.60
Middle SDI	11,909,532.71	8,230,217.13	3,679,315.58	17,078,127.92	11,771,978.72	5,306,149.19
Low-middle SDI	4,210,209.62	2,729,444.99	1,480,764.63	9,543,180.02	6,188,506.22	3,354,673.80
Low SDI	8,496,266.55	5,616,814.74	2,879,451.81	14,051,074.93	9,265,382.96	4,785,691.97
Andean Latin America	262,167.08	171,034.84	91,132.24	455,159.81	301,417.12	153,742.70
Australasia	233,589.06	162,909.80	70,679.26	357,327.29	250,609.73	106,717.56
Caribbean	247,324.01	161,213.90	86,110.11	320,814.51	210,958.46	109,856.05
Central Asia	625,275.75	400,093.42	225,182.33	858,328.07	556,913.91	301,414.16
Central Europe	1,155,443.60	743,600.92	411,842.68	1,055,437.25	689,527.87	365,909.38
Central Latin America	1,275,975.93	835,224.87	440,751.06	1,917,270.63	1,258,615.03	658,655.61
Central Sub-Saharan Africa	507,732.42	325,716.61	182,015.81	1,281,660.80	828,522.75	453,138.04
East Asia	7,670,293.15	5,741,450.58	1,928,842.57	9,470,370.32	7,203,852.35	2,266,517.97
Eastern Europe	2,016,643.85	1,265,248.05	751,395.80	1,828,389.10	1,158,891.92	669,497.17
Eastern Sub-Saharan Africa	1,782,238.92	1,142,732.80	639,506.12	4,016,784.62	2,587,097.57	1,429,687.05
High-income Asia Pacific	2,475,304.43	1,726,827.74	748,476.69	2,681,977.52	1,877,978.81	803,998.72
High-income North America	2,964,103.19	1,985,609.58	978,493.61	3,892,297.01	2,629,485.82	1,262,811.19
North Africa and Middle East	2,667,504.57	1,766,204.04	901,300.53	4,884,140.26	3,285,875.32	1,598,264.94
Oceania	46,426.63	31,871.63	14,554.99	96,966.05	66,638.33	30,327.72
South Asia	7,795,864.25	5,230,719.69	2,565,144.56	12,848,944.99	8,570,781.41	4,278,163.58
Southeast Asia	3,201,991.31	2,094,066.33	1,107,924.97	4,784,649.86	3,186,798.30	1,597,851.55
Southern Latin America	516,198.42	349,935.64	166,262.78	700,574.69	477,938.97	222,635.72
Southern Sub-Saharan Africa	488,295.87	310,267.45	178,028.42	741,682.65	475,865.37	265,817.28
Tropical Latin America	960,545.40	627,420.95	333,124.45	1,381,082.91	908,897.68	472,185.23
Western Europe	3,247,150.17	2,359,184.46	887,965.71	3,672,369.02	2,691,540.86	980,828.16
Western Sub-Saharan Africa	1,789,927.79	1,149,607.02	640,320.77	4,577,312.27	2,915,670.92	1,661,641.35

SDI, Socio-demographic Index.

**Table 2.** **Age-Standardized Prevalence Rate (ASPR) of ASD per 100,000 population by location and sex in 1990 and 2021, with Estimated Annual Percentage Change (EAPC) from 1990 to 2021**.

Location	ASPR			ASPR			ASPR		
	1990			2021			EAPC (%)		
	Both	Male	Female	Both	Male	Female	Both	Male	Female
Global	773.25	1046.01	497.32	788.34	1064.71	508.08	0.07	0.06	0.09
High SDI	1038.81	1439.57	637.53	1056.99	1457.49	642.78	0.06	0.05	0.03
High-middle SDI	777.19	1077.89	476.56	796.49	1113.47	466.58	0.11	0.13	–0.016
Middle SDI	669.36	909.52	420.84	703.96	957.3	444.05	0.17	0.17	0.22
Low-middle SDI	698.66	909.15	480.58	716.95	939.56	491.98	0.08	0.10	0.07
Low SDI	789.11	1020.3	555.5	809.1	1052.31	567.28	0.08	0.10	0.06
Andean Latin America	663.53	872.17	458.33	684.26	902.45	464.45	0.10	0.12	0.04
Australasia	1162.13	1619.24	703.54	1191	1670.02	709.43	0.08	0.11	0.03
Caribbean	687.39	907.64	472.9	682.49	902.69	464.73	–0.01	–0.01	–0.05
Central Asia	876.88	1143.25	624.79	886.03	1154.37	621.48	0.05	0.05	–0.004
Central Europe	934.71	1214.86	659.1	964.38	1263.25	662.27	0.11	0.14	0.008
Central Latin America	745.71	989.74	509.14	758.59	1017.12	510.3	0.06	0.08	0.006
Central Sub-Saharan Africa	865.32	1126.41	613.89	885.37	1152.02	624.5	0.07	0.07	0.05
East Asia	618.25	897.95	321.24	660.67	972.17	324.56	0.24	0.26	0.16
Eastern Europe	906.09	1188.78	644.76	928.54	1226.18	645.58	0.09	0.12	0.007
Eastern Sub-Saharan Africa	874.61	1136.74	619.09	893.46	1165.9	628.95	0.07	0.08	0.046
High-income Asia Pacific	1442.14	2014.92	870.01	1559.53	2161.06	938.79	0.25	0.23	0.23
High-income North America	1072.08	1450.08	699.68	1097.16	1486.93	707.32	0.10	0.11	0.062
North Africa and Middle East	755.54	978.08	521.84	771.8	1001.27	524.85	0.07	0.08	0.021
Oceania	678.31	901.66	438.74	673.2	898.18	433.53	–0.03	–0.02	–0.04
South Asia	681.98	881.92	465	686.16	896.99	466.77	0.01	0.05	0.0015
Southeast Asia	663.62	875.37	456.79	682.98	905.87	458.63	0.10	0.11	0.028
Southern Latin America	1035.24	1425.59	657.8	1056.55	1458.73	662.33	0.05	0.05	0.017
Southern Sub-Saharan Africa	890.22	1169.17	631.75	903.63	1184.91	637.32	0.06	0.05	0.027
Tropical Latin America	608.68	803.85	418.67	614.52	820.69	413.38	0.03	0.06	–0.032
Western Europe	876.83	1280.61	473.68	896.6	1309.14	477.59	0.09	0.09	0.041
Western Sub-Saharan Africa	879.6	1138.9	618.82	886.16	1162.87	626.93	0.03	0.07	0.046

SDI, Socio-demographic Index; ASPR, Age-Standardized Prevalence Rate; ASD, Autism Spectrum Disorder; EAPC, Estimated Annual Percentage Change.

### SDI Level

**Fig. 3.**
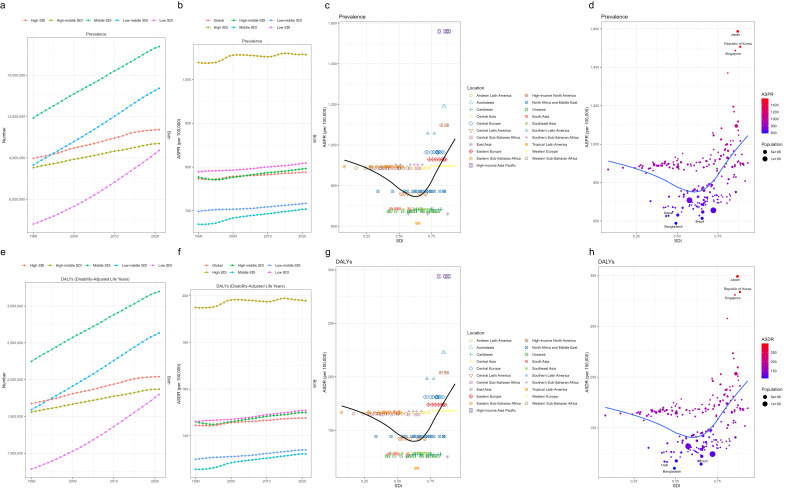
**Distribution and temporal trends of ASD-related prevalence, DALYs, ASPR, and ASDR by 5 SDI regions, 21 GBD regions, and 204 countries/territories**. Number of prevalence and DALYs by 5 SDI groups from 1990 to 2021 (a,e); Global and 5 SDI ASPR and ASDR trends from 1990 to 2021 (b,f); ASPR and ASDR by SDI across 21 GBD regions in 2021(c,g); ASPR and ASDR by SDI across 204 countries and territories in 2021(d,h). ASPR, Age-Standardized Prevalence Rate; ASDR, Age-Standardized Disability-Adjusted Life Years Rate; GBD, Global Burden of Disease; DALYs, Disability-Adjusted Life Years; ASD, Autism Spectrum Disorder; SDI, Socio-demographic Index.

From 1990 to 2021, the EAPC for ASPR and ASDR varied across SDI regions (Fig. 2a,b; Table 2, **Supplementary Table 2**). The Middle SDI region showed the most significant increase, with an EAPC for ASPR of 0.17 (95% CI: 0.16 to 0.18) and an EAPC for ASDR of 0.18 (95% CI: 0.17 to 0.19). In contrast, the High SDI region had the slowest increase, with an EAPC for ASPR of 0.064 (95% CI: 0.048 to 0.079) and an EAPC for ASDR of 0.057 (95% CI: 0.041 to 0.073). In both 1990 and 2021, the <5 age group exhibited the highest prevalence and DALY rates across nearly all SDI regions. A notable exception occurred in the High SDI region in 1990, where the highest prevalence rate was observed in the 15 to 19 age group (Fig. 2c–f). Regarding the absolute burden, the number of prevalent cases and DALYs in all five SDI regions showed a continuous upward trend from 1990 to 2021, with the Middle SDI region having the highest number of prevalent cases and DALYs, while the Low SDI region had the lowest (Fig. 3a,e). In terms of age-standardized rates, the High SDI region consistently had the highest ASPR and ASDR among the five SDI regions throughout the period, while the Middle SDI region had the lowest (Fig. 3b,f).

### 21 GBD Region Level

From 1990 to 2021, most of the 21 GBD regions showed an upward trend in ASPR and ASDR, with only the Caribbean and Oceania regions showing a downward trend (Fig. 2a,b; Table 2;** Supplementary Table 2)**. The most pronounced increases occurred in high-income Asia Pacific, with an EAPC of 0.25 for both ASPR and ASDR. In contrast, Oceania showed the most marked decreases, with an EAPC of –0.027 for ASPR and –0.018 for ASDR (Fig. 2a,b; Table 2;** Supplementary Table 2**). In both 1990 and 2021, the <5 age group had the highest prevalence and DALYs rates in all 21 GBD regions, while the 95 plus age group had the lowest, consistent with the global trend (Fig. 2c–f). In 2021, among the 21 GBD regions, the ASPR and ASDR showed a trend of first decreasing and then increasing with higher SDI levels. The highest ASPR and ASDR were observed in high-income Asia Pacific, while the lowest were recorded in Tropical Latin America (Fig. 3c,g). Detailed numerical estimates are provided in the Table 2 and **Supplementary Table 2**.

### Country Level

**Fig. 4.**
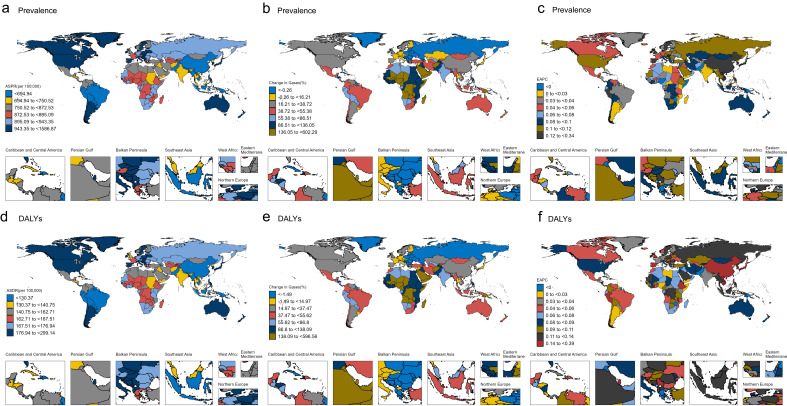
**Global spatial distribution, temporal relative changes, and epidemiological trend analysis of ASD-related indicators (1990–2021)**. Global maps of ASD-ASPR and ASD-ASDR in 2021 (a,d), changes in prevalence and DALYs cases from 1990 to 2021(b,e), and EAPC of prevalence and DALYs from 1990 to 2021(c,f). ASD, Autism Spectrum Disorder; ASPR, Age-Standardized Prevalence Rate; ASDR, Age-Standardized Disability-Adjusted Life Years Rate; DALYs, Disability-Adjusted Life Years; EAPC, Estimated Annual Percentage Change.

In 2021, the ASPR and ASDR in 204 countries and territories globally showed a trend of first decreasing and then increasing with higher SDI levels (Fig. 3d,h). Meanwhile, there were significant differences in prevalent cases and DALYs (Fig. 4a,d). Japan, the Republic of Korea, and Singapore represented the three countries with the highest ASPR and ASDR. The lowest ASPR estimates were observed in Bangladesh, Brazil, and Nepal, while the lowest ASDR values were recorded in Bangladesh, Brazil, and Haiti (**Supplementary Tables 3,4**). From 1990 to 2021, there were significant variations in the changes in the number of prevalent cases and DALYs globally (Fig. 4b,e). Qatar had the largest increase in the number of prevalent cases and DALYs, with a 602.29% increase in the number of cases and a 596.56% increase in DALYs compared to 1990. Georgia had the largest decrease in the number of prevalent cases and DALYs, with a 36.59% decrease in the number of cases and a 37.29% decrease in DALYs compared to 1990 (**Supplementary Tables 5,6**). From 1990 to 2021, there were also significant variations in the changes in ASPR and ASDR globally (Fig. 4c,f). Similarly, ASPR and ASDR trends varied markedly across countries (Fig. 4c,f). Equatorial Guinea recorded the most pronounced increase in both ASPR (EAPC = 0.34) and ASDR (EAPC = 0.39), while Zimbabwe showed the greatest decline in these metrics (EAPC = –0.092 for both). Detailed numerical estimates are provided in the **Supplementary Tables 3,4**.

### Decomposition Analysis

**Fig. 5.**
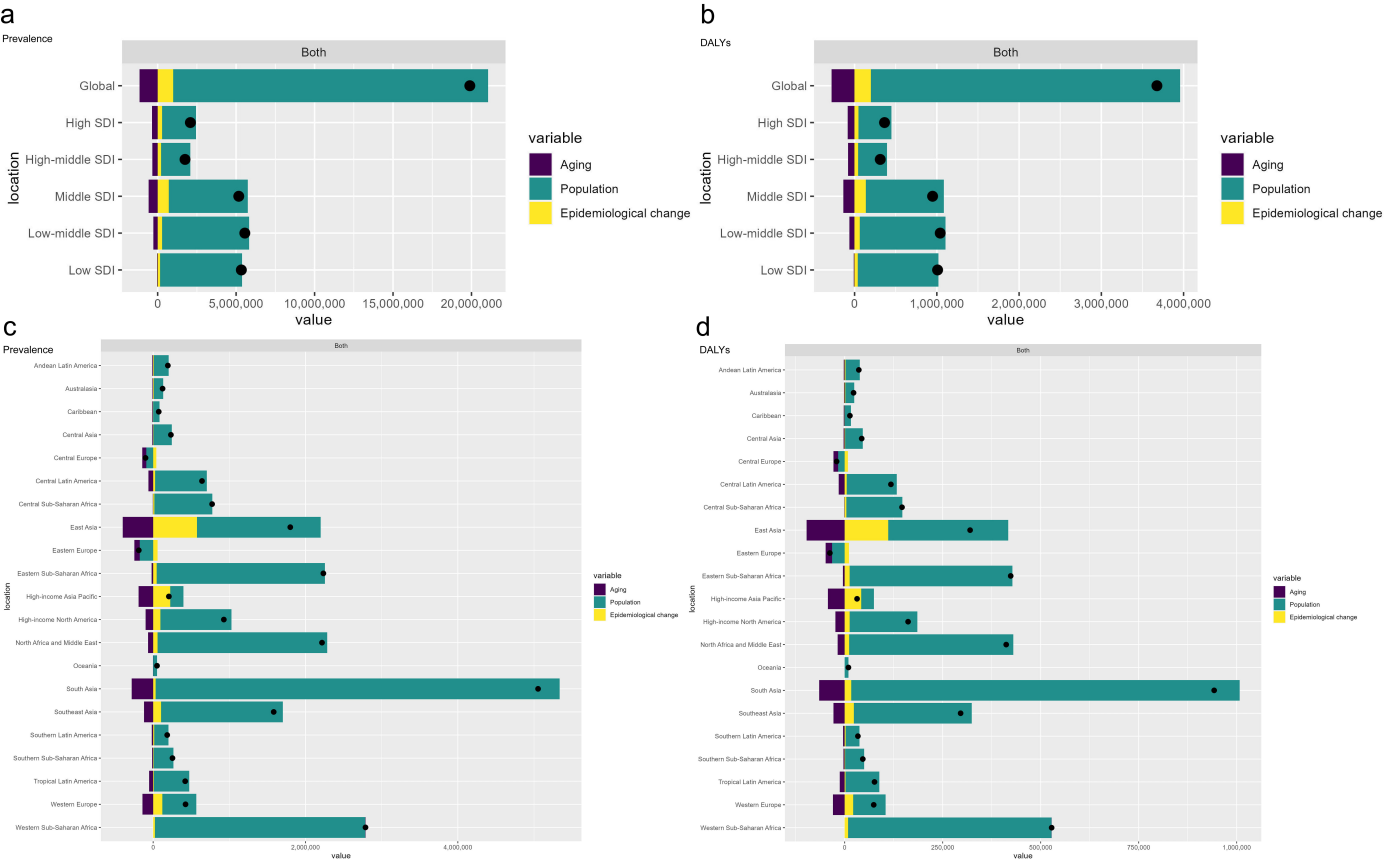
**Decomposition Analysis of ASD Burden Changes (1990–2021) across Global, SDI, and GBD Regional Levels**. Decomposition analysis of ASD prevalence (a,c) and DALYs (b,d) across global and five SDI regions (a,b), as well as 21 GBD regions (c,d). ASD, Autism Spectrum Disorder; SDI, Socio-Demographic Index; GBD, Global Burden of Disease; DALYs, Disability-Adjusted Life Years.

We used decomposition analysis to assess the impact of aging, population growth, and epidemiological changes on the number of prevalent cases and DALYs globally, across five SDI regions, and across 21 GBD regions (Fig. 5). From 1990 to 2021, globally and across the five SDI regions, as well as in most of the 21 GBD regions, the number of prevalent cases and DALYs showed a significant increase, except for Central Europe and Eastern Europe, where the number of prevalent cases and DALYs decreased. Globally and across the five SDI regions, population growth was the main driver of the increase in the number of prevalent cases and DALYs, while aging was the main factor contributing to the decrease (Fig. 5a,b). From 1990 to 2021, in the Oceania region, population growth was the main driver of the increase in the number of prevalent cases, while epidemiological changes were the main factor contributing to the decrease. In the high-income Asia Pacific region, epidemiological changes were the main driver of the increase, while aging was the main factor contributing to the decrease. In Central Europe and Eastern Europe, population decline was the main driver of the decrease, while epidemiological changes were the main factor contributing to the increase. In Western Sub-Saharan Africa, all three factors contributed to the increase, with population growth being the main driver. In other regions, population growth was the main driver of the increase in the number of prevalent cases, while aging was the main factor contributing to the decrease (Fig. 5c). For DALYs, in the high-income Asia Pacific region, epidemiological changes were the main driver of the increase, while aging was the main factor contributing to the decrease. In Central Europe and Eastern Europe, population decline was the main driver of the decrease, while epidemiological changes were the main factor contributing to the increase. In Western Sub-Saharan Africa, all three factors contributed to the increase, with population growth being the main driver. In other regions, population growth was the main driver of the increase in DALYs, while aging was the main factor contributing to the decrease (Fig. 5d). Detailed numerical estimates are provided in the** Supplementary Tables 7,8**.

### Nordpred Model

**Fig. 6.**
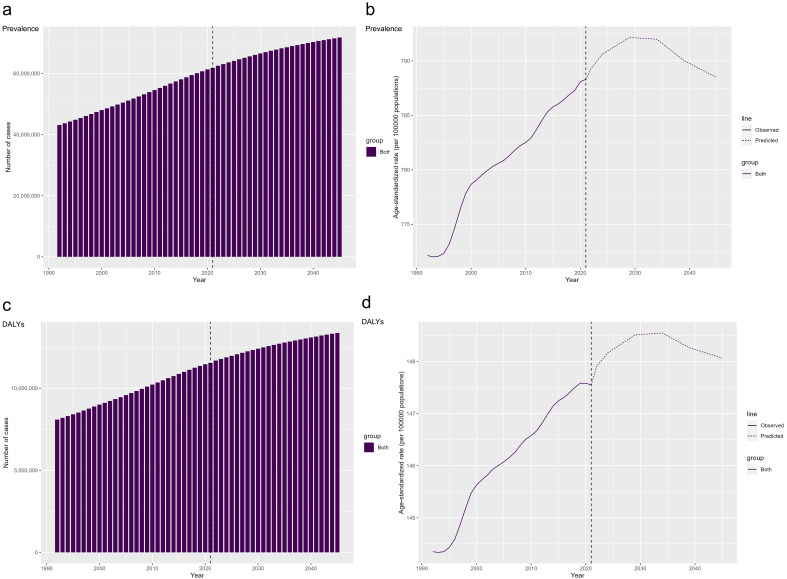
**Projected Global Burden of ASD: Prevalence, DALYs, ASPR, and ASDR from 1992 to 2045**. Global ASD Forecasts: Number of Prevalence (a), ASPR(b), Number of DALYs (c), and ASDR (d). ASD, Autism Spectrum Disorder; DALYs, Disability-Adjusted Life Years; ASPR, Age-Standardized Prevalence Rate; ASDR, Age-Standardized Disability-Adjusted Life Years Rate.

We used the Nordpred model to predict the trends in the number of prevalent cases, DALYs, ASPR, and ASDR for ASD globally until 2045 (Fig. 6). It is predicted that after 2021, the number of prevalent cases and DALYs will continue to increase steadily, showing a continuous upward trend. By 2045, the global number of prevalent cases is expected to reach approximately 71,782,946, and DALYs are expected to reach approximately 13,365,467 years (Fig. 6a,c). The ASPR and ASDR are expected to increase initially and then decline after 2021. The ASPR is expected to peak at 792.16 cases per 100,000 in 2029 before gradually declining, while the ASDR is expected to peak at 148.55 years per 100,000 in 2034 before gradually declining (Fig. 6b,d) Detailed numerical estimates are provided in the **Supplementary Tables 9,10**.

## Discussion

This study, based on the latest GBD 2021 data, provides a comprehensive analysis of the epidemiological characteristics and burden of ASD across global, SDI-specific, regional, and national levels. Utilizing advanced methodologies including EAPC, decomposition analysis, and the Nordpred prediction model, we assessed temporal trends, quantified drivers of disease burden, and projected future trajectories of ASD.

Our analysis revealed several key findings regarding ASD burden and demographic disparities. Globally, ASD prevalent cases and ASPR rose steadily from 1990 to 2021, paralleled by increases in DALYs and ASDR. Although multiple studies have confirmed the upward trend in ASD prevalence [23, 24], the interpretation of this increase remains debated. Twin cohort studies have found no evidence of change over time in the genetic and environmental factors underlying ASD and autistic traits, suggesting that shifts in environmental factors alone cannot account for the observed increase [25]. Furthermore, a study by Sebastian Lundström *et al*. [26] found that while clinical ASD diagnoses in Swedish children rose significantly, the underlying autistic symptom phenotypes remained stable over time. This supports the interpretation that the recorded increase in ASD prevalence may be more attributable to improved administrative oversight, refinements in diagnostic criteria and instrumentation, and earlier and more frequent diagnosis, rather than a true rise in incidence. Our study found that the burden of ASD is significantly higher in males than in females, with a male-to-female ratio of approximately 2:1 for incidence, prevalence, and DALYs. This differs from the 3:1 ratio estimated in GBD 2019, possibly due to changes in GBD inclusion criteria, which excluded some cases reliant on passive reporting, or an increased recognition of underdiagnosis in females [27]. Previous studies have suggested a larger gender disparity in ASD, potentially as high as 4:1, but growing evidence indicates that this ratio may be exaggerated, with underdiagnosis of females being a significant issue. Females with ASD are more likely to have comorbid conditions, exhibit more subtle symptoms, and face potential gender biases in diagnostic methods, all of which may contribute to the underestimation of ASD prevalence in females [28]. Our study found a slight narrowing of the gender disparity in ASD, as the increase in prevalence among females was more pronounced than in males, consistent with findings by Zhen Li *et al*. [27]. This trend may be related to the growing attention to female autism and the increasing detection rate [29, 30]. Both in 1990 and 2021, the prevalence and DALYs rates of ASD decreased with age, with the highest disease burden observed in children under five years old. However, it is important to note that autism is a lifelong condition, and with the aging population, identifying and managing ASD in adulthood and old age presents new challenges [31, 32].

Significant regional and socioeconomic variations in ASD burden were observed. Significant disparities in ASPR and ASDR for ASD were observed across SDI regions, GBD regions, and countries. The Middle SDI region experienced the largest increase in ASD burden, while the High SDI region showed the slowest upward trend. These variations are closely linked to differences in economic development, healthcare access, diagnostic capacity, and public awareness of ASD [33]. In High SDI regions, advanced healthcare systems and widespread early screening may have slowed the rise in ASD burden, whereas in Middle SDI regions, improved diagnostic capabilities and population growth drove significant increases [34, 35]. Notably, Low SDI regions had the lowest number of prevalent cases and DALYs, likely reflecting insufficient diagnostic capabilities, incomplete data recording, and limited healthcare resources rather than true lower prevalence [35]. Among the 21 GBD regions, high-income Asia Pacific showed the most significant increase in ASPR and ASDR, while Oceania showed a declining trend, potentially resulting from effective public health interventions and widespread early screening programs [34, 35]. Furthermore, in 2021, ASPR and ASDR showed a nonlinear relationship with SDI levels, with the highest burden in high-income Asia Pacific and the lowest in Tropical Latin America. At the country level, Japan, South Korea, and Singapore had the highest ASPR and ASDR values, while Bangladesh, Brazil, and Nepal had the lowest. From 1990 to 2021, Qatar showed the largest increase in prevalent cases and DALYs, while Georgia experienced the largest decrease. Equatorial Guinea and Zimbabwe showed the most extreme changes in ASPR and ASDR. Aderinto *et al*. [36] attribute such leaps to weak past detection in Africa—better tools, awareness and healthcare now reveal hidden burden, not new epidemics.

Through decomposition analysis, we identified key contributors to the trends in ASD burden. This study employed decomposition analysis to break down the number of prevalent cases and DALYs into different influencing factors, such as aging, population growth, and epidemiological changes, providing a more detailed understanding of these trends. The results indicate that among the Global, five SDI regions, and 21 GBD regions, only Central Europe and Eastern Europe showed improvements in disease burden, while the burden increased in other regions. Population growth was identified as the primary driver of changes in disease burden in most regions. As the population grows, the number of prevalent cases and DALYs also increases. This suggests that even if the prevalence of ASD remains relatively stable, the overall disease burden will rise significantly due to population expansion. Additionally, the impact of epidemiological changes on disease burden varied by region. In some regions, epidemiological changes contributed to an increase in disease burden, while in others, they helped mitigate it. For example, in the High-income Asia Pacific region, the increase in disease burden was primarily driven by the widespread adoption of diagnostic criteria and improvements in healthcare resources. The highly developed healthcare systems in this region, particularly in countries like Japan, South Korea, and Singapore [37, 38, 39], have facilitated early screening programs and the use of advanced diagnostic tools such as the Autism Diagnostic Observation Schedule, Second Edition (ADOS-2) and the Autism Diagnostic Interview-Revised (ADI-R). This has significantly improved case identification and reporting rates. Moreover, increasing awareness of ASD among the public and healthcare professionals has reduced social stigma and encouraged more families to seek diagnosis and support. These factors collectively contributed to a significant rise in diagnostic rates, leading to a noticeable increase in disease burden in this region. Aging, on the other hand, played a mitigating role in disease burden in most regions. However, this statistical association requires critical interpretation. A simplistic demographic explanation—that an aging population reduces the proportion of children—is inadequate. Instead, the apparent mitigation may reflect a statistical artifact, akin to the‘diagnostic substitution’phenomenon observed in special education data, where increases in ASD identification coincide with decreases in other diagnostic categories [40]. More importantly, this perspective is inconsistent with the established understanding of ASD as a lifelong condition that presents unique challenges in later life [41, 42]. Research indicates that older autistic adults face significant health disparities, including higher rates of physical and mental health comorbidities and a markedly reduced life expectancy compared to the general population [41]. Therefore, the aging process does not reduce the burden but unveils a previously hidden and growing need among an aging ASD population, for whom healthcare and social support systems are critically underprepared [42].

Projections from the Nordpred model indicate complex future trajectories for ASD burden. Our analysis of global data from 2022 to 2045 projects a steady increase in the number of prevalent cases and DALYs, reflecting a persistent upward trend in absolute burden. In contrast, the ASPR and ASDR are projected to peak in 2029 and 2034, respectively, followed by a gradual decline. This divergent trend likely stems from countervailing factors: on one hand, increased prevalence of ASD, improved diagnostic tools (e.g., standardized use of ADOS-2), and heightened public awareness have collectively enhanced case identification [43, 44]; on the other hand, global population aging, the optimization of diagnostic standards and healthcare systems, and broader socioeconomic improvements are expected to eventually stabilize and then reduce the standardized rates of diagnosis. Together, these forces shape the projected transition in the global epidemiology of ASD.

Policy recommendations for resource-limited settings. Addressing the global ASD burden requires context-specific strategies, particularly in low-resource settings that face well-documented challenges in early identification, including significantly delayed age of ASD diagnosis [45]. Beyond the recommended low-cost screening tools (M-CHAT, STAT, CAST) and training for primary providers [34, 46], implementation should include: (1) task-shifting strategies using non-specialist health workers for initial screening and family support; (2) community-based rehabilitation programs leveraging local resources; and (3) mobile health technologies for remote assessment and training. The WHO’s “Global Autism Action Plan” should prioritize funding for these approaches in low-SDI regions, focusing on community diagnostic centers and family interventions [47]. Public education should expand beyond awareness campaigns to include neurodiversity-focused school programs and workplace inclusion initiatives [48]. Research should emphasize implementation science to translate gene-environment interaction findings into practical interventions [49], while international networks like INSAR must specifically support data collection and intervention studies in underrepresented regions [50].

This study has several important limitations that warrant consideration. First, the decomposition analysis, while useful for quantifying the contributions of population growth, aging, and epidemiological changes, operates under the assumption that these factors are independent; it may not fully capture their complex interactions or synergistic effects on ASD burden. Second, the use of EAPC for trend analysis assumes a linear change over time, which may not fully capture more complex, non-linear epidemiological transitions in ASD burden. Third, the Nordpred prediction model, while widely used, relies on historical data patterns and stable population structures; significant demographic shifts or unforeseen changes in diagnostic practices could affect the accuracy of long-term projections. Fourth, GBD data reliability depends on national health systems; underreporting in developing countries may underestimate ASD burden. Fifth, model-based ASD estimates risk errors in data-scarce regions, as assumptions may oversimplify diagnostic/cultural complexities. Finally, limited spatiotemporal resolution in GBD data hinders local/short-term trend accuracy, impacting intervention planning.

## Conclusion

In conclusion, this study highlights a significant and increasing global burden of ASD, with notable variations across regions, genders, and age groups. The rising trend is driven by a combination of factors, including population growth, improved diagnostics, and increasing awareness. Future projections suggest a continued increase in the number of prevalent cases and DALYs, while the ASPR and ASDR are expected to peak in 2029 and 2034, respectively, before gradually declining. Addressing this challenge requires a multifaceted approach, including early intervention, equitable resource allocation, public education, and ongoing research.

## Availability of Data and Materials

The data that support the findings of this study are available at https://vizhub.healthdata.org/gbd-results/.

## Supplementary Material

Supplementary material associated with this article can be found, in the online version, at https://doi.org/10.62641/aep.v53i6.2029.
