# Supplementary material for: Global, Regional, and National Burden of Autism Spectrum Disorder: Trends and Decomposition Analysis From 1990 to 2021, and Projections for 2045
Source: Actas Esp Psiquiatr. 2025 Dec 17;53(6):1162–75. doi: 10.62641/aep.v53i6.2029 (PMC12728558; doi:10.62641/aep.v53i6.2029)
Supplement: Supplementary file 1 [file ActEsp-53-6-1162-1175-s1.zip › Supplementary+Figure+and+Table+Legend.docx]

**Supplementary Figure Legend**

**Supplementary Fig. 1:** Estimated Annual Percentage Change (EAPC) in Age-Standardized Prevalence Rate (ASPR) and Age-Standardized Disability-adjusted Life Years Rate (ASDR) for autism spectrum disorder from 1990 to 2021, by sex and location.

Panels show EAPC values for ASPR in males (a) and females (b), and for ASDR in males (c) and females (d). Locations are ordered by EAPC magnitude within each panel. EAPC values represent the average annual percentage change in rates over the study period.

Abbreviations: EAPC, Estimated Annual Percentage Change; ASPR, Age-Standardized Prevalence Rate; ASDR, Age-Standardized Disability-adjusted Life Years Rate.

**Supplementary Table:**

**Supplementary Table 1:** Number of Disability-Adjusted Life Years (DALYs) of autism spectrum disorder (ASD) by location and sex in 1990 and 2021.

**Supplementary Table 2:** Age-Standardized DALYs Rate (ASDR) of ASD per 100,000 population by location and sex in 1990 and 2021, with Estimated Annual Percentage Change (EAPC) from 1990 to 2021.

**Supplementary Table 3:** ASPR of ASD per 100,000 population across 204 countries and territories by sex in 1990 and 2021.

**Supplementary Table 4:** ASDR of ASD per 100,000 population across 204 countries and territories by sex in 1990 and 2021.

**Supplementary Table 5:** Number of prevalent cases of ASD across 204 countries and territories by sex in 1990 and 2021.

**Supplementary Table 6:** Number of DALYs of ASD across 204 countries and territories by sex in 1990 and 2021.

**Supplementary Table 7:** Decomposition analysis of changes in prevalent cases of autism spectrum disorders from 1990 to 2021 by region.

**Supplementary Table 8:** Decomposition analysis of changes in DALYs of autism spectrum disorders from 1990 to 2021 by region.

**Supplementary Table 9:** Projected global number of prevalent cases and ASPR of ASD from 2022 to 2045 by sex.

**Supplementary Table 10:** Projected global number of DALYs and ASDR of ASD from 2022 to 2045 by sex.

Abbreviations: ASD, autism spectrum disorder.; ASDR, Age-Standardized DALYs Rate; EAPC, Estimated Annual Percentage Change;
